# Extending Shelf Life of Indonesian Soft Milk Cheese (Dangke) by Lactoperoxidase System and Lysozyme

**DOI:** 10.1155/2018/4305395

**Published:** 2018-05-31

**Authors:** Ahmad Ni'matullah Al-Baarri, Anang Mohamad Legowo, Septinika Kurnia Arum, Shigeru Hayakawa

**Affiliations:** ^1^Department of Food Technology, Faculty of Animal and Agricultural Sciences, Diponegoro University, Semarang 50275, Indonesia; ^2^Laboratory of Food Technology, Integrated Laboratory, Diponegoro University, Semarang 50275, Indonesia; ^3^Department of Animal Science, Faculty of Animal and Agricultural Sciences, Diponegoro University, Semarang 50275, Indonesia; ^4^Department of Applied Biological Sciences, Faculty of Agriculture, Kagawa University, Miki-cho 761-0795, Japan

## Abstract

Dangke, a type of fresh soft cheese made of bovine and buffalo milk, is a traditional dairy product used in South Sulawesi, Indonesia. It is prepared from fresh milk using the conventional method, which easily destroys the quality. This study was conducted to assess whether using lactoperoxidase system and lysozyme as preservative agents could suppress the growth of bacteria in dangke. The pH value, total microbial count, and hardness of dangke were determined to measure the quality. Lactoperoxidase and lysozyme were purified from fresh bovine milk, and their purity was confirmed using SDS-PAGE. The combination of lactoperoxidase system and lysozyme was able to remarkably suppress the total microbial count in dangke from 7.78 ± 0.67 to 5.30 ± 0.42 log CFU/ml during 8 h of storage at room temperature. Preserving dangke in this enzyme combination affected its hardness, but there was no remarkable change in the pH value. Results of this study may provide knowledge to utilize a new method to preserve the quality of dangke.

## 1. Introduction

Dangke, a type of fresh soft cheese, is a traditional dairy product available in Enrekang Regency, South Sulawesi province, Indonesia. The nutrient content of dangke in %w/w comprises 55% water, 23.8% protein, 14.8% fat, and 2.1% ash [1]. It is produced by heating fresh milk and then adding papaya latex to precipitate casein. Commonly, local people used papaya latex from unripe papaya fruit, thus keeping the slightly bitter taste [2]. Traditionally, curd and whey are separated using a coconut shell, which is a process involved in the shaping stage in the preparation of dangke. After the shaping process, dangke is packed in a banana leaf and is ready to be consumed. The conventional method of producing dangke does not involve high food hygiene standards, resulting in an increased possibility for contamination with bacteria. Dangke is usually preserved using salt, though there is the problem of a relatively short shelf life (±2 days) by storing at room temperature [1].

Today, the production of dangke has increased along with the increase in consumer demand [2]. The distribution of dangke has been reported to reach out of the province, including to other countries such as Brunei Darussalam and Malaysia. Nationally, dangke is being already distributed to Java and Sumatra islands, consistent with the increase in the number of tourism activities. Therefore, preservation is an important factor to maintain the quality of dangke.

Lactoperoxidase (LPO) is a heme-containing glycoprotein of 608 amino acids with a molecular mass of 78 kDa and has already been known as a natural enzyme found in plants, animals, and humans. LPO is abundantly found in milk, saliva, and tear glands [3–5] and can serve as a natural antimicrobial in combination with thiocyanate (SCN^−^) and hydrogen peroxide (H_2_O_2_), which is known as the lactoperoxidase system (LPOS) [5–7]. LPO catalyzes the oxidation of thiocyanate (SCN^−^) by hydrogen peroxide (H_2_O_2_), resulting in the production of hypothiocyanite (OSCN^−^). Hypothiocyanite is a compound that is responsible for killing bacteria, fungi, and viruses by destroying the sulfhydryl groups (SH groups) of the cell membrane, resulting in damage to the vital cell membrane, which leads to cell death [8–12]. LPOS has been used as a natural preservative in some foods, such as milk [13, 14], fruits, chicken, and vegetables [15]. LPOS is effective in suppressing the growth of* Pseudomonas*,* Escherichia coli*, and* Salmonella typhimurium* on cottage cheese [16].

Lysozyme (1,4-beta-N-acetylmuramidase, 14.4 kDa) is a hydrophilic protein that has been widely used as a natural preservative. It is naturally found in egg white and milk [17, 18]. Lysozyme hydrolyzes 1,4-*β*-linkages between N-acetylmuramic acid and N-acetylglucosamine present in the peptidoglycan. Gram-positive bacteria are highly susceptible to lysozyme because of the presence of peptidoglycan in their cell walls, but lysozyme is not effective in killing Gram-negative bacteria [19–21], which indicates the need for a combination with other compounds. Lysozyme has been used as an antimicrobial and an antiviral in food and pharmaceutical industries [22], where it causes inhibition of the growth of pathogenic bacteria and could thus extend the shelf life of food. It is also used in the preservation of fruits, vegetables, beans, tofu, curd, meat, sausages, salads, and semi-hard-type cheese such as Edam, Gouda, and some Italian cheese. It has also been reported to have protective effects against pathogenic bacteria such as* Bacillus cereus* in cheese [23]. On the other hand, lysozyme has also been added to infant formulas to achieve the similarity to human milk [24, 25].

Previous research has shown that weak inhibition by LPOS in dangke could result in the extension of shelf life for only 6 h at room temperature [2]. Therefore, a synergistic effect of LPOS to inhibit bacteria may be useful to solve this problem. Thus, in this study, lysozyme was added to LPOS to extend the shelf life of dangke. This experiment might provide knowledge to utilize a new method for extending the shelf life of dangke using natural LPOS and lysozyme.

## 2. Materials and Methods

### 2.1. Materials

Fresh bovine milk samples were provided by a campus farm. Fresh duck eggs were purchased from a local farm. Latex from young papaya was used to obtain papain enzyme to precipitate the protein. SP-Sepharose Fast Flow (SP-FF) (Lot No. 10072021) was used for lysozyme purification. LPO from bovine whey was obtained from the Chemical and Food Nutrition Laboratory, Food Technology Department, Faculty of Animal and Agricultural Sciences, Diponegoro University. Hydrogen peroxide (H_2_O_2_) and potassium thiocyanate (KSCN) were used as LPO substrate. A 0.2 *μ*m syringe filter was used to sterilize the enzyme.

### 2.2. Lysozyme Purification

Lysozyme purification was carried out following the method described by Naknukool et al. [26]. Duck egg white was mixed with 3-fold volume sodium acetate buffer (0.05 M, pH 5.0). The mixture was centrifuged at 6000 rpm for 15 min to separate the supernatant, and then the supernatant was applied in an SP-FF column for lysozyme purification. Then, 500 ml of sodium acetate buffer (0.05 M, pH 5.0) was subsequently eluted through the column. Lysozyme was obtained using serial dilution with 300 ml of 0.1, 0.3, and 0.5 M NaCl in sodium phosphate buffer (0.05 M, pH 9.0). The eluate was then collected in 10 ml tubes. The purity of the eluate was determined using sodium dodecyl sulfate polyacrylamide gel electrophoresis (SDS-PAGE).

### 2.3. Preparation of Dangke

The preparation of dangke was adopted from the traditional method that has been followed by the local people in South Sulawesi. Fresh bovine milk was heated at 75°C for 20 min, and then latex from young papaya (0.3% w/v) was subsequently added to the milk. The curd that formed was separated using a clean filter cloth and then pressed to produce dangke. Using this method, 680 g of dangke could be produced from 1 l of milk.

### 2.4. Preparation of LPOS Solution

LPOS solution was prepared using a mixture of 300 *μ*L of LPO, 300 *μ*L of 0.9 mM H_2_O_2_, and 300 *μ*L of 0.9 mM KSCN. Prior to application, this mixture was filtered using a 0.2 *μ*m syringe filter, placed in a microtube, and left to stand for 1 h at 30°C.

### 2.5. Preservation of Dangke

A total of 1 g of dangke was used for the evaluation of total microbial count and the pH value, while for the evaluation of hardness, dangke was cut into a rectangular shape measuring 2.5 × 1 × 1 cm. The dangke was then stored at 30°C for 18 h for the calculation of total microbial count, while the dangke stored at 30°C for 8 h was used to analyze the pH value and hardness. Prior to evaluation, dangke was immersed in various preservation solutions (LPOS, lysozyme, and LPOS + lysozyme) at 30°C for 4 h. Dangke immersed in sterile pure water was used as a control.

### 2.6. Microbial Count

3 M Petrifilm Aerobic Count Plates (3 M Microbiology, St. Paul, Minn., USA) were used for assessing the microbial count of dangke following a previous method described by Rasbawati [2], with a minor modification. Briefly, dangke was subjected to serial dilutions of sterile 0.88% NaCl solution to enumerate the bacteria. The diluted mixture (1000 *μ*l) was spread onto the plates and incubated at 37°C for 48 h. The CFUs of the microbes in the sample solution were counted on the plates.

### 2.7. Hardness Measurement

Dangke samples measuring 2.5 × 1 × 1 cm were analyzed for hardness. Texture analyses were conducted using Brookfield Texture Analyzer (CT3) under the following conditions: a ø12.7 mm ball probe was penetrated to a depth of 4 mm into the sample at a speed of 1 mm/s, and the textural hardness was measured in triplicate and expressed in Newton.

### 2.8. Statistical Analysis

The total microbial count was analyzed descriptively with two replications. The pH value and hardness were analyzed using ANOVA with three replications. Statistical analyses were performed using R software for Macintosh. Duncan's multiple range test (*P* < 0.05) was used to calculate the significance among values.

## 3. Results and Discussion

### 3.1. Purification of Lysozyme

**Figure 1:**
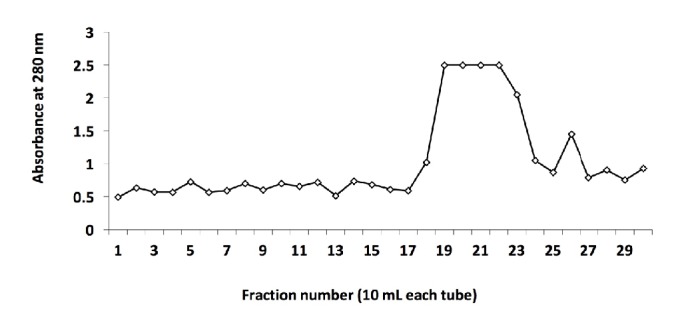
Absorbance at 280 nm of the eluate from SP Sepharose Fast Flow column (10 ml each tube) containing a high concentration of protein. Fraction numbers 1–10, 11–20, and 21–30 were obtained from elution with 0.1, 0.3, and 0.5 M NaCl in sodium phosphate buffer (0.05 M, pH 9.0), respectively.

**Figure 2:**
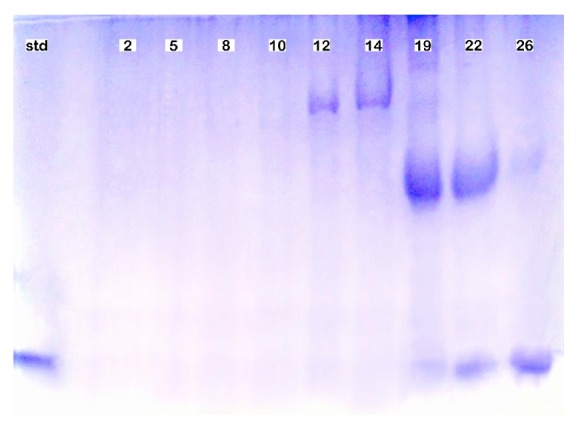
Sodium dodecyl sulfate polyacrylamide gel electrophoresis (SDS-PAGE) profile of eluate through SP Sepharose Fast Flow. Lane from left to right: standard (std) using *α*-lactalbumin (a 14 kDa protein), fraction numbers 2, 5, 8, 10, 12, 14, 19, 22, and 26. Fraction number 26 showed a single band, indicating that pure lysozyme was detected. Thus, this fraction was used for the entire research.

Three-step dilutions with various concentrations of NaCl were carried out to obtain the lysozyme.  Figure 1 shows the absorbance at 280 nm of the elution from each step of dilution. Fractions numbers 1–10, 11–20, and 21–30 were obtained from the elution against phosphate buffer (pH 9.0) containing 0.1, 0.3, and 0.5 M NaCl, respectively. A high peak of protein concentration activity was detected from fractions numbers 19–22. However, the elution from these fractions showed more than one band (Figure 2), whereas fraction number 26 showed a single band representing pure lysozyme with a molecular weight of 14 kDa. Therefore, fraction number 26 was used for the entire study. The elution was then mixed, and the protein concentration was determined using the Lowry method, resulting in a value of 0.10%. This value was comparable to that reported in another study that showed that the protein concentration from purified protein determined using a similar method was almost 0.1% [25].

### 3.2. Total Microbial Count

**Figure 3:**
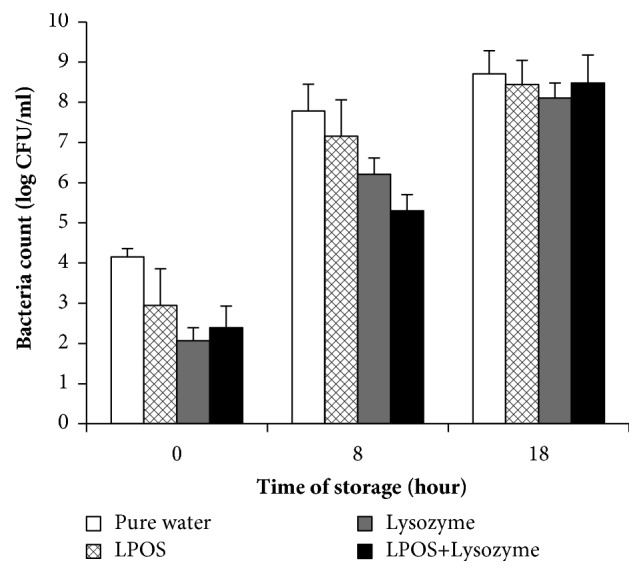
Total microbial count in dangke after immersing for 10 min in solutions containing LPOS, lysozyme, and the combination of LPOS + lysozyme. Dangke immersed in pure water was used as control. Values are the mean from three replicates of the experiment, and error bars represent standard error.

Figure 3 shows the total microbial count in the dangke samples that were immersed in sterile pure water, LPOS, lysozyme, and a combination of LPOS + lysozyme for 18 h at room temperature. It can be seen that the total microbial count in dangke has increased by storage time. Immersing in sterile pure water at 0 h showed the highest bacterial count (4.15 ± 0.21 CFU/ml) compared to those with other treatments, whereas immersing dangke in lysozyme resulted in the lowest total number of bacteria (2.07 ± 0.32 log CFU/ml). Immersing dangke in LPOS and the combination of LPOS + lysozyme resulted in a total bacterial number of 2.95 ± 0.91 and 2.39 ± 0.54, respectively. Immersing dangke for 8 h increased the total bacterial count in all treatments, ranging from 5.30 ± 0.42 to 7.78 ± 0.67 log CFU/ml, and a longer immersion time of up to 18 h resulted in further increase in the bacterial count, ranging from 8.11 ± 0.37 to 8.71 ± 0.57 log CFU/ml (Figure 3).

Immersing dangke in LPOS and lysozyme or its combination reduced the total microbial count, as shown in Figure 3. LPOS, lysozyme, and the combination of LPOS + lysozyme were able to decrease the population of bacteria at 0 h of storage to almost 1.20, 2.08, and 1.76 log CFU/ml, respectively, when compared to the total bacterial count in dangke immersed in sterile pure water as control. Among all the treatments, lysozyme exhibited the strongest antibacterial activity, whereas LPOS exhibited the weakest antimicrobial activity.

The antibacterial activity of LPO is due to hypothiocyanite production from the enzymatic reaction between hydrogen peroxide and thiocyanate. Hypothiocyanite is a short-lived product that is responsible for killing bacteria, fungi, and viruses by destructing the sulfhydryl (SH) groups of the cell membrane [3, 8, 26–28]. Lysozyme is known to exert its antimicrobial activity against bacteria, fungi, protozoa, and viruses by destroying the structural components on the cell walls of bacteria and fungi [29–31]. Lysozyme catalyzes the *β*1–4 bonds between N-acetylmuramic acid and N-acetylglucosamine in the peptidoglycan, resulting in bacterial death. Gram-positive bacteria are highly susceptible to lysozyme as they contain 90% peptidoglycan in their cell walls, whereas the peptidoglycan content in Gram-negative bacteria is only 5%–10% [19, 32]. It has been well documented that several bacteria found in raw milk might also be found in cheese due to the handling process prior to cheese-making [33]. Among these bacteria, the Gram-positive bacteria such as* Enterococcus*,* Pediococcus*,* Aerococcus*,* Staphylococcus* [33], and* Bacillus* spp. [34] are commonly found in milk. The dominance of Gram-positive bacteria may provide an answer for the high antimicrobial activity of lysozyme in cheese.

In the present study, all the preservatives were unable to inhibit the growth of bacteria in dangke stored for 18 h because of the high total microbial count (from 8.11 ± 0.37 to 8.71 ± 0.57 log CFU/ml). This result is consistent with [35] that showed that the hypothiocyanite generated from limited amount of substrates (0.3 mM H_2_O_2_ and 0.3 mM SCN^−^) was able to kill the total bacteria in milk if the initial population of bacteria did not exceed 8.00 log CFU/ml. Furthermore, [2] reported that the LPOS was unable to reduce the total microbial count in dangke stored for 12 h with a total microbial count of 10^10^ CFU/ml.

The combination LPOS + lysozyme was unable to suppress the growth of bacteria in dangke at the maximum storage time; however, the synergistic effect of this combination could be observed at 8 h of storage of dangke, resulting in the least total bacterial count of 5.30 ± 0.42 CFU/ml compared to that with other treatments. Since the Indonesian National Standard (2008) has stated that the maximum allowed limit of total bacteria in cheese is 6 log CFU/ml, the combination of LPOS + lysozyme may be applied to meet the requirement of the maximum allowed amount of total bacteria in cheese.

### 3.3. pH Value

The development of appropriate pH and texture is required to produce the preferred cheese by storage during a period of time [36]. Based on the data shown in Table 1, the pH value of dangke stored at room temperature for 8 h varied from 6.22 ± 0.30 to 6.77 ± 0.02. Dangke immersed in sterile pure water showed a significant increase in pH value, ranging from 6.22 ± 0.30 to 6.54 ± 0.05, whereas immersing dangke in LPOS, lysozyme, and the combination of LPOS + lysozyme did not show a significant change in the pH value.

It has been reported that the increase in pH value was due to the process of deamination of amino acids resulting in the production of NH_3_ and the metabolism of lactic acid bacteria to produce CO_2_ [37]. This reason is in agreement with the result of total bacteria shown in Figure 3, where the total bacterial count was found to be decreased along with treatments in the preservative solutions. The decreased number of live bacteria contributed to the decreased production of CO_2_, resulting in less change in the pH value.

The initial pH value of dangke was detected to be 6.22 ± 0.30, while [2] stated that the initial pH value of dangke was 7.17. Another study reported an initial pH value of 6.40 [38]. It has been recognized that the initial pH value of dangke was relatively similar to the pH of fresh milk. The variation in the initial pH value of dangke may be explained by the wide variation in the pH value of papaya latex. It has been documented that the pH of papaya latex ranged from 6.00 to 8.75 [38, 39], thus probably resulting in the alteration of initial pH value of dangke from the initial pH value of fresh milk.

### 3.4. Hardness

Table 2 shows the results of the measurement of hardness of dangke immersed in sterile pure water, LPOS, lysozyme, and the combination of LPOS + lysozyme at 0 h of storage time (initial) and 8 h of storage time (final). Based on the statistical analysis, sterile pure water and the combination of LPOS + lysozyme had no significant effects on the hardness of dangke; however, LPOS increased the hardness of dangke to a value of 71.6% from the initial point, resulting in final textural hardness of 3.62 ± 0.90 N. The hardness of dangke immersed in lysozyme was found to be significantly decreased. Based on the results shown in Table 2, the decrease in hardness of dangke immersed in lysozyme was 36%, resulting in a final hardness value of 1.750 ± 0.32 N. The increase in hardness of dangke immersed in LPOS may be explained by the generation of hypothiocyanite and hypothiocyanous acid by the enzymatic reaction between KSCN and H_2_O_2_ using LPO as a catalyzer. Reference [40] stated that hypothiocyanite is an anion and the conjugate base of hypothiocyanous acid which is an organic compound and a part of thiocyanate containing the functional group SCN^−^. Hypothiocyanous acid is a fairly weak acid with an acid dissociation constant of 5.3 [41]. It has been recognized that some factors, including pH, can affect the rheological properties of dangke. For instance, a decrease in pH of Gouda cheese resulted in an increase in hardness [42] and vice versa, which is similar to the result of the present study.

The measurement of hardness is necessary to determine the quality of rheological properties. Since dangke is commonly consumed after deep frying or is served with other food products, a hard-texture-dangke is commonly preferred. Therefore, based on this reason, the LPOS treatment might be an appropriate method to preserve dangke and strengthen its hardness.

## 4. Conclusions

LPOS, lysozyme, and the combination of LPOS + lysozyme were able to inhibit the growth of microbes in dangke stored for 8 h. The highest antimicrobial activity was found in dangke preserved in the combination of LPOS + lysozyme immersion. The change in pH value was also maintained by immersing dangke in all treatments. The hard texture of dangke was found in dangke immersed in LPOS; therefore the treatment with the combination of LPOS and lysozyme was suggested to retain the softness of dangke.

## Figures and Tables

**Table 1:** pH value of dangke immersed in pure water, LPOS, lysozyme, and the combination of LPOS + lysozyme.

Storage period (h)	Dangke pH value			
	Pure water	LPOS^ns^	LZ^ns^	LPOS + LZ^ns^
0	6.22 ± 0.30^b^	6.59 ± 0.01	6.72 ± 0.01	6.71 ± 0.01
1	6.46 ± 0.13^a^	6.48 ± 0.09	6.54 ± 0.04	6.62 ± 0.01
2	6.54 ± 0.06^a^	6.64 ± 0.07	6.71 ± 0.01	6.62 ± 0.01
3	6.52 ± 008^a^	6.68 ± 0.10	6.72 ± 0.05	6.64 ± 0.05
4	6.43 ± 0.03^ab^	6.56 ± 0.11	6.71 ± 0.03	6.64 ± 0.08
5	6.54 ± 0.05^a^	6.75 ± 0.07	6.69 ± 0.03	6.71 ± 0.08
6	6.53 ± 0.04^a^	6.64 ± 0.05	6.73 ± 0.09	6.68 ± 0.03
7	6.46 ± 0.02^a^	6.58 ± 0.11	6.69 ± 0.01	6.64 ± 0.02
8	6.54 ± 0.05^a^	6.66 ± 0.01	6.77 ± 0.02	6.70 ± 0.03

The superscript letters indicate significant difference among the storage periods; "ns" means not significant. Data are the average values from triplicate of the experiment ± standard error.

**Table 2:** Hardness (N) of dangke after immersing in pure water, LPOS, lysozyme, and the combination of LPOS + lysozyme.

Dangke	Pure water^ns^	LPOS	LZ	LPOS + LZ^ns^
Initial	1.984 ± 0.75	2.110 ± 0.56^b^	2.734 ± 0.47^a^	2.035 ± 0.69
Final	1.535 ± 1.03	3.620 ± 0.90^a^	1.750 ± 0.32^b^	2.798 ± 0.73

The superscript letters indicate significant difference among the storage periods; "ns" means not significant. Data are the hardness values at 8 h storage at 30°C.

## Data Availability

The data used to support the findings of this study are available from the corresponding author upon request.
